# Effects of Pilates Training Combined with Fascial Massage on Upper Cross Syndrome in Office Workers

**DOI:** 10.3390/healthcare13040410

**Published:** 2025-02-14

**Authors:** Liao Jiang, Yada Thadanatthaphak, Kukiat Tudpor

**Affiliations:** 1 Department of Health and Sport Science, Faculty of Education, Mahasarakham University, Maha Sarakham 44150, Thailand; 63010565005@msu.ac.th; 2 Faculty of Public Health, Mahasarakham University, Maha Sarakham 44150, Thailand; kukiat.t@msu.ac.th

**Keywords:** upper cross syndrome, Pilates training, fascial massage, electromyography, cervical spine dysfunction

## Abstract

**Objective:** Upper crossed syndrome (UCS) is an abnormal upper extremity movement pattern characterized by muscle tightness in the neck, shoulders, and upper back, coupled with weakness in opposing muscle groups. This study aimed to investigate the effectiveness of Pilates training combined with fascial massage as an intervention in office workers with UCS. **Methods:** 34 subjects were recruited and randomly divided into an experimental group (n = 17) and a control group (n = 17). The control group underwent 12 weeks of Pilates training, and the experimental group received 12 weeks of Pilates training combined with fascial massage. Body posture was assessed using the forward head angle (FHA) and forward shoulder angle (FSA), joint mobility was evaluated using cervical spine range of motion (ROM), muscle activity was assessed using surface electromyography (sEMG), and quality of life was evaluated using pain level (VAS) and cervical spine dysfunction index (NDI). **Results:** After 12 weeks of intervention, the FHA, FSA, VAS, and NDI of the experimental group were significantly lower than those of the pre-intervention group (*p* < 0.05) and significantly lower than those of the control group (*p* < 0.05); the extension and left–right rotation cervical spine ROM of the experimental group were significantly higher than those of the pre-intervention group (*p* < 0.05) and significantly higher than those of the control group (*p* < 0.05); and sEMG indexes (mean power frequency and median frequency) of the upper trapezius and the pectoralis major in the experimental group were significantly higher than those of the pre-intervention group (*p* < 0.05) and significantly higher than the control group (*p* < 0.05). **Conclusion:** Compared with Pilates training alone, Pilates training combined with fascial massage demonstrated a more significant effect in improving muscle activation, body posture, and pain and enhancing the quality of life for patients with UCS.

## 1. Introduction

Upper crossed syndrome (UCS) is characterized by specific altered muscle activation, movement patterns, and postural deviations [1]. Its characteristics include tension in the upper trapezius (UT) and pectoralis major muscles (PM1), as well as weakness in the lower trapezius (LT), all located in the neck, shoulders, and upper back [2]. Previous studies have shown the prevalence of UCS at 11–60% in different social and age groups [3], and it is one of the prevalent postural disorders in society. According to Nasser et al., 2021, the prevalence of UCS was 24.32% among drivers, 27.03% among homemakers, 37.1% among medical students, and as high as 32.43% among office workers [4]. This may be because office populations are sedentary for long periods and lack regular activity, which can easily lead to tension and imbalance of weak muscle groups and abnormal postures such as crooked heads, hunched backs, and rounded shoulders. Studies have shown that when muscles hold a posture or perform repetitive movements for a long period of time, it strengthens the tonic system (single-jointed muscles) and weakens the tensor system (multi-jointed muscles), which leads to muscle imbalance and triggers abnormal postures such as head tilting (increased anterior curvature of the cervical vertebrae), hunching (increased posterior curvature of the thoracic vertebrae), and rounding of the shoulders (increased abduction of the shoulder blades) [5].

Additionally, UCS can be accompanied by various physical ailments and symptoms. Some studies have shown that UCS causes patients to have a forward shift in the center of gravity, leading to reduced balance, susceptibility to falls, and accidental injuries, and accompanied by headaches, shoulder and neck pain, chronic muscle injuries, decreased lung capacity, and limited joint mobility, which not only seriously affects the quality of life, but also has a particular impact on socioeconomic development [6]. Therefore, exploring options to improve this abnormal movement pattern of UCS is crucial.

Currently, rehabilitation treatments for UCS include exercise therapy, postural correction, muscle stretching, etc. For example, Karimian et al. conducted a comparative study of 12 UCS faculty members at the National College of Sports Medicine. They found that after 12 weeks of self-myofascial release, stretching, and strengthening exercises, the experimental group significantly reduced forward head posture, shoulder angle, and kyphosis compared to a control group that did not participate in the program [7]. Bayattork et al. performed an 8-week exercise correction on 24 male UCS patients. They found that the level of trapezius activation, as measured by surface electromyography, significantly improved after implementing a corrective exercise program compared to a control group that did not participate [8]. In addition, Aneis et al. employed a multimodal approach for 20 patients with UCS, encompassing ergonomically recommended posture correction exercises, muscle energy techniques, cervical stabilization exercises, and scapulothoracic stabilization exercises [9]. They compared the outcomes with 20 UCS patients who underwent only muscle energy techniques and found that the multimodal approach was more effective than the single rehabilitation method.

Pilates training is a comprehensive form of physical exercise characterized by its combination of unique movements, precise breath control, and core strengthening. Chusan Yin found that Pilates effectively reduced pain, improving shoulder pronation and cervical disability index compared to conventional exercise therapy [10]. Zeng found that a 12-week Pilates intervention effectively reduced shoulder and neck pain, improved cervical dysfunction, and enhanced cervical joint mobility in UCS patients [11]. However, few empirical studies exist on using Pilates for interventions in UCS [11].

In recent years, fascial massage, a therapeutic maneuver that can effectively improve muscle adhesions, relieve pain, and help muscles regain normal physiological properties, has also received increasing attention in treating UCS. Several studies have examined the role of fascial massage on body posture and found that it helps with neuromuscular conditioning and mechanical relaxation, restores the balance of muscular forces, promotes the formation of good body posture, and prevents sports injuries. Ruivo et al. conducted a study on 15- to 17-year-old adolescents who underwent fascial stretching over 8 months and found that it was effective in helping adolescents with symptoms of head forward tilt to alleviate their head forward tilt symptoms [12].

Incorporating Pilates as a therapeutic means for UCS might be practical and feasible. However, no research has been combining Pilates training with fascial massage to explore its impact on UCS populations. Therefore, this study integrates fascial massage into Pilates training and conducts a 12-week exercise intervention targeting UCS populations among office workers to investigate its effectiveness for UCS. Our hypothesis is as follows: Pilates training alone and Pilates training combined with fascial massage can improve UCS in office workers, but Pilates training combined with fascial massage is more effective.

## 2. Materials and Methods

### 2.1. Study Design

**Figure 1:**
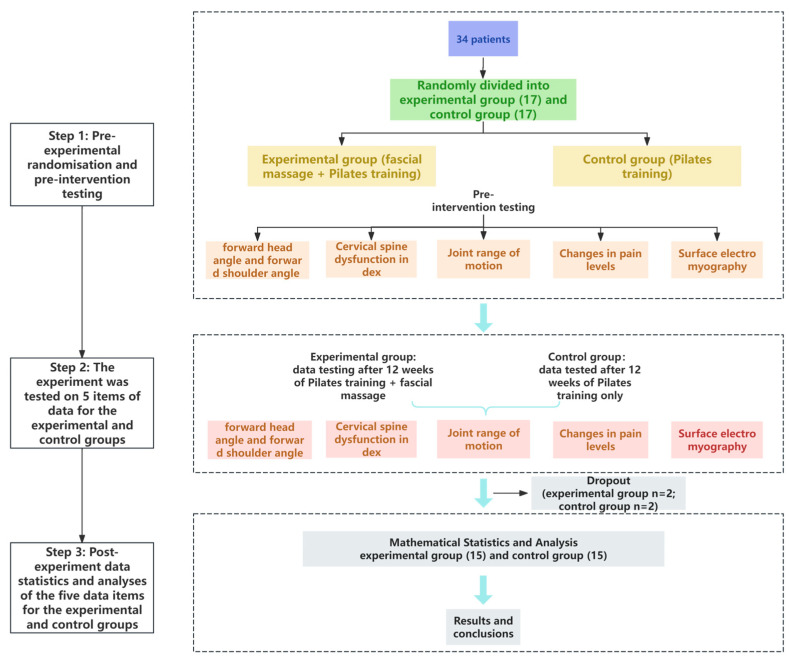
The experimental flow.

The study was a single-blind, randomized controlled trial, using a random number table method to divide participants into two groups. An experimental group included Pilates training combined with fascial massage, and a control group included Pilates training alone. The experimental flow is shown in Figure 1.

### 2.2. Ethical Consideration

This study was conducted under the approval of the Ethics Committee of Mahasarakham University. Approval number: 275-242/2024.

### 2.3. Participants

This study’s volunteers were recruited online and offline through WeChat, Sina Weibo, 58 Tongcheng, and other platforms, as well as offline through public welfare lectures, posters, and flyers. The public lectures were held at the Sichuan Health and Rehabilitation Vocational College, the Rehabilitation Center of Zigong No. 4 People’s Hospital, and the Rehabilitation Department of Zigong No. 1 People’s Hospital. Recruited volunteers were included in the trial after systematic assessment by a team of professional rehabilitation therapists and specialized physicians who met the following inclusion-exclusion criteria.

Inclusion Criteria: (1) aged between 25 and 60 years old; (2) without cognitive impairment; (3) with obvious somatic problems, such as round shoulders, hunchback, FHA ≥ 46°, FSA ≥ 52°; (4) patients with some degree of discomfort such as neck and shoulder pain, stiffness, muscle tightness, etc., and accompanied by limited neck and shoulder movement; (5) no congenital cervical spondylolisthesis, bone tumor, bone tuberculosis or other bone and joint diseases; (6) voluntarily participated and voluntarily signed the informed consent, and were able to complete the examination and training as required.

Exclusion criteria: (1) have apparent degenerative lesions of cervical intervertebral disks, history of neck and shoulder trauma or surgery; (2) have severe cardiac, hepatic, renal or pulmonary insufficiency, bone tumors, bone tuberculosis, pregnant women, and postoperative dysfunctions; (3) those who have undergone other treatments or taken part in other sports within 3 months.

Based on previous studies and considering the attrition rate of the sample, we ultimately included 34 volunteers [13,14], and they were randomly and equally divided into a test group (17) and a control group (17). Each volunteer was informed of the specific experimental objectives and procedures but was unaware of their group assignment. Before the study, all participants completed an informed consent form and were informed of their right to withdraw from the study at any time without penalty. Two subjects in the control group did not have post-test data collected because they did not wish to perform the postintervention test, one was excluded from the intervention group because of less than 75% attendance, and one did not perform the postintervention test because of a scheduling conflict, for a total of four subjects who were excluded because they did not meet the data criteria. Ultimately, 30 cases were included in the data analysis (experimental group n = 15; control group n = 15).

### 2.4. Intervention

Seven experts with extensive experience were interviewed before the experiment, including all frontline participants and researchers with a long history of rehabilitation medicine and athletic training. They combined the characteristics of Pilates training and UCS to design a targeted Pilates training program for the subjects [14,15,16]. After determining the asanas in Pilates, the duration of asana practice, the number of times different asanas were practiced, and the intervals between Pilates training, a team of professional Pilates instructors and rehabilitation therapists conducted a 12-week experimental intervention on the experimental group and the control group in the Pilates training room. The experimental group had a fascial massage before Pilates training, then performed Pilates training 3 times a week for 1 h each time. The control group only performed Pilates training 3 times a week for 1 h. The specific intervention program is shown in Table 1.

### 2.5. Test Indicators

In this study, professional rehabilitation therapists conducted static postural assessments and screenings. The volunteers’ forward scapular angle (FSA) and forward head angle (FHA) were measured using a grid diagram and a plumb line for body posture evaluation [17]. The FHA is a standard indicator for evaluating resting neck posture in UCS patients, with clinical validity. An FHA of 36°or more indicates the presence of abnormal cervical curvature. FSA refers to the angle formed by the shoulders bending forward. The semicircular arc formed by the shoulders is known as rounded shoulders, and the angle between this semicircular arc and the body’s centerline is defined as the FSA. FSAs are semicircular arcs formed by bending the shoulders forward. When the shoulder crest moves forward (relative to the spinous process of the 7th cervical vertebra), scapular anteversion and kyphosis symptoms are manifested as round shoulders. This study measured the FHA and the FSA using the Static Postural Assessment and Postural Screening System. The measurement method is as follows: make a vertical line along the seventh cervical vertebra and the horizontal plane, and then take the seventh cervical vertebra as the starting point and connect the line to the ear screen and the angle formed by the vertical line and the connecting line from the seventh cervical vertebra to the ear screen was the FHA; then make a vertical line along the seventh cervical vertebra and the horizontal plane, and then take the seventh cervical vertebra as the starting point and connect the line to the crest of the shoulder, and the angle formed by the vertical line and the connecting line from the seventh cervical vertebra to the crest of the shoulder was the FHA. The FSA is the angle formed by the vertical line and the line connecting the seventh cervical vertebra to the acromion.

Changes in the cervical range of motion (ROM) reflect the function of the cervical spine [18]. Before and after the intervention, a professional rehabilitation therapist measured the ROM of the cervical joints in flexion and extension, left and right lateral flexion, and left and right rotation using a square, circular disk goniometer.

The Neck Disability Index (NDI), a validated questionnaire assessing neck function [19], consists of 10 self-report questions covering daily activities, attention, and pain. The NDI covers activities of daily living (7 dimensions), attention (1 dimension), and pain (2 dimensions). Each question has six response options, with a maximum score of 5 and a minimum score of 0. It will be employed to evaluate neck function in UCS patients. A specialized rehabilitation therapist conducted assessments before and after the intervention.

The visual analog scale (VAS), a simple measure of pain intensity in clinical practice, is the most acceptable, simple, and least misunderstood scale for objectively scoring changes in pain levels and providing a more objective evaluation of the effectiveness of pain management [20]. Its basic method of assessment is to use a 10 cm line with 11 scales with a “0” end and a “10” end, with a score of 0 representing no pain and a score of 10 representing the most intense pain, and the farther to the right the scale the subject examines, the farther to the right the scale is.

Surface electromyography (sEMG) captures bioelectrical signals during movement, reflecting neuromuscular activity and functional level. This was tested by a trained professional rehabilitation therapist. In this study, firstly, the sEMG signals of the UCS population were collected with the help of a ME6000 telemetric sEMG tester; secondly, the collected data were converted into Fourier’s spectrum by the software MegaWin version 1.0, and the parameter values of mean power frequency (MPF) and median frequency (MF) were calculated for each muscle, which can intuitively reflect the changes in muscle function (e.g., fatigue resistance) in the UCS population and are an essential basis for evaluating the effects of the intervention. The overactive PM1, UT, and LT were selected as test muscles [21].

First, soak a medical swab with 75% medical alcohol; second, wipe the skin surface of the test muscle with the soaked medical swab; finally, after the skin surface of the test muscle is sufficiently dry, attach the electrodes along the direction of the muscle. For the selection of electrode placement locations, reference was made to the body surface localization of commonly used surface EMG electrodes in the book Clinical Application of Surface Electromyography Diagnostic Techniques, edited by Li Jianhua and Wang Jian [22]. Position of UT electrodes: first, determine the midpoint of the line connecting the seventh cervical vertebrae to the crest of the shoulder, and then place the two electrodes in order on the left and right side of the midpoint, with the two electrodes 1 cm apart, and place the reference electrode 2 cm below the midpoint of the two electrodes. The reference electrode was placed 2 cm below the midpoint of the two electrodes; the position of the PM1 electrode sheet on the chest wall is about 6 cm below the clavicle. The position of the LT electrode sheet: first, determine the midpoint of the 10th thoracic vertebrae facing 45 degrees to the upper right to the lower corner of the scapula toward the spine, then place the two electrodes sequentially on the left and the right of the midpoint, with the two electrodes 1 cm apart, and place a reference electrode 2 cm below the midpoint of the two electrodes. A reference electrode was placed 2 cm below the midpoint of the two electrodes.

Maximal voluntary contraction tests were performed on each of the three muscles; before the test, the subjects were allowed to complete a 10 min preparatory activity. The EMG signals of each muscle were recorded for 10 s of maximal voluntary isometric contraction (MVIC) while the subject was performing the test maneuver. Each muscle was tested 3 times, with a 30 s rest between each test, and the average of the three tests was used as a calibration value. After each muscle was tested, there was a 2 min rest before testing the next muscle. UT: The subject was asked to sit in a chair, look straight ahead, and place their feet flat on the floor, then abduct the left shoulder and raise the scapula for 10 s while the person in charge of the evaluation was asked to apply a resistance above the subject’s shoulder; the same was carried out for the right trapezius superior fasciculus test. PM1: To test the left pectoralis major muscle, the subject was asked to lie down on a cushion and then perform a right shoulder flexion test. Then, the subject flexed the right shoulder joint horizontally and held it for 10 s. At the same time, the person in charge of the assessment applied a resistance to the left with their hand on the palm of the subject’s hand; when testing the right pectoralis major, the subject’s cervical spine activity is in the opposite direction, and the other matters are the same. LT maximal voluntary contraction test method: when testing the left trapezius infraspinatus, the subject first lay flat on a mat, raised their arms to be either side of their head, and was then asked to perform a downward press on the scapula and hold it for 10 s, while the person in charge of the assessment applied resistance on the proximal humerus with their hand. The same is performed when testing the right trapezius infraspinatus muscle.

Post-intervention testing of the indicators was completed within 3–5 days after the last intervention. It is worth noting that before and after the intervention, all the tests were performed by the same rehabilitation therapist to ensure consistency and comparability of the assessment process. In other words, although different rehabilitation therapists can perform different tests, the same rehabilitation therapist is responsible for assessing the same test before and after the intervention.

### 2.6. Data Analysis

Data from the pre-test and post-test of the experiment were analyzed using SPSS 26.0. Descriptive statistics were calculated using mean ($\bar{X}$) and standard deviation (SD) for continuous variables. A normality test was conducted on the collected data to determine whether the data conformed to a normal distribution (*p* < 0.05). Before and after the intervention, those that conformed to normal distribution were explored for between-group differences using the independent samples *t*-test; paired *t*-tests were used to assess within-group changes over time.

## 3. Results

### 3.1. Basic Information

The independent samples *t*-test was conducted based on the baseline information of the pre-intervention characteristics of the two groups of subjects, and the results are shown in Table 2. There was no significant difference in the baseline information of age, height, weight, and BMI between the two groups, and they were homogeneous.

### 3.2. FHA and FSA Results

**Figure 2:**
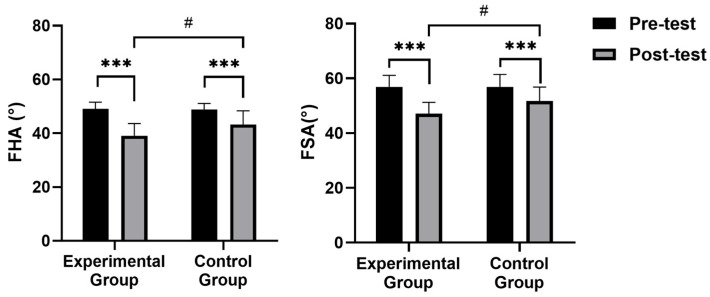
Comparison of forward head angle (FHA) and forward shoulder angle (FSA). Note: *** significant difference within groups (*p* < 0.001); # significant difference between groups (*p* < 0.05).

Forward head angle (FHA) and forward shoulder angle (FSA) results of the within-group and between-group comparisons are shown in Figure 2. There was no significant difference (*p* > 0.05) in FHA and FSA between the participants in both groups before the intervention. After the intervention, FHA and FSA in the experimental group were significantly lower than those before the intervention (*p* = 0.000 and 0.002). Moreover, FHA and FSA in the experimental group were significantly lower than in the control group (*p* = 0.026 and 0.011).

### 3.3. Cervical ROM Results

**Figure 3:**
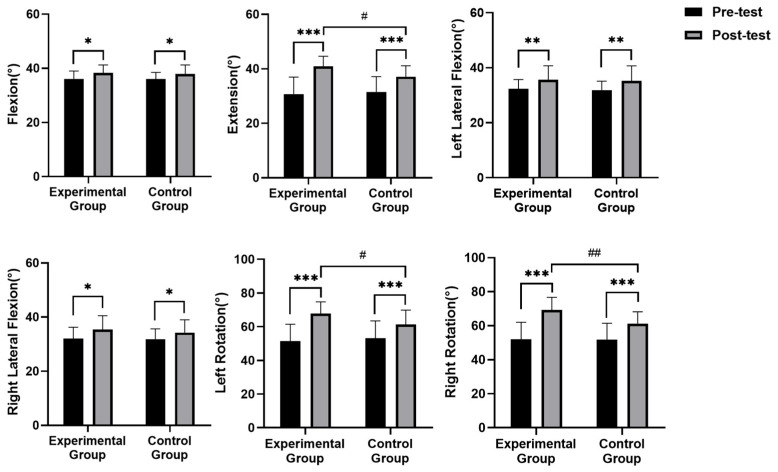
Comparison of cervical range of motion. Note: * significant difference within groups (*p* < 0.05); ** significant difference within groups (*p* < 0.01); *** significant difference within groups (*p* < 0.001); # significant difference between groups (*p* < 0.05); ## significant difference between groups (*p* < 0.01).

Figure 3 shows that, before the intervention, no statistically significant difference was observed in the range of motion (ROM) for flexion and extension between the two groups (*p* > 0.05). A substantial increase in both flexion (*p* = 0.027) and extension (*p* = 0.000) ROM was observed in the experimental group. The control group also significantly improved flexion (*p* = 0.025) and extension (*p* = 0.000) ROM. Significant increases were observed in both left (*p =* 0.001) and right (*p =* 0.017) lateral flexion ROM in the experimental group. The control group also significantly enhanced left (*p* = 0.002) and right (*p* = 0.024) lateral flexion ROM. Both left (*p* = 0.000) and right (*p* = 0.000) rotation ROM significantly improved in both groups. No significant group differences were found in flexion and extension ROM (*p* = 0.720), although the experimental group had significantly greater extension ROM (*p* = 0.012). No significant group differences were observed in lateral flexion ROM before or after the intervention (*p* > 0.05). No significant group differences were observed at baselines in left and right rotation ROM (*p* > 0.05). However, post-intervention, the experimental group exhibited significantly greater left (*p* = 0.030) and right (*p* = 0.005) rotation ROM than the control group.

### 3.4. sEMG Results

**Figure 4:**
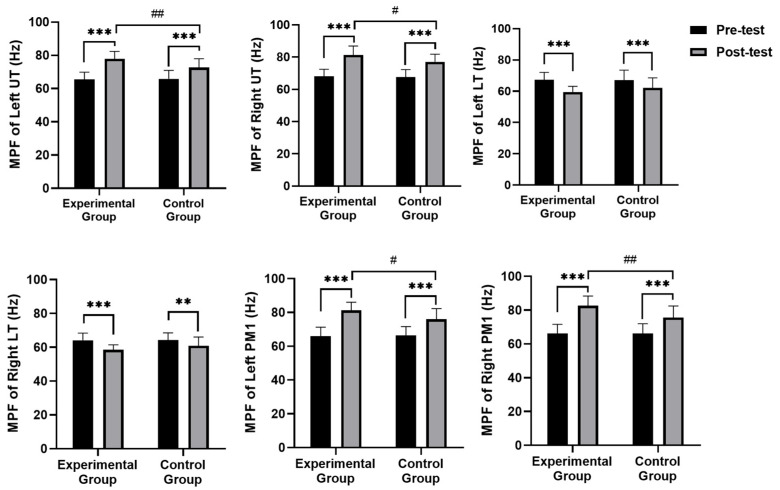
Comparison of frequency domain metrics mean power frequency (MPF) of surface electromyography (sEMG). Note: UT—upper trapezius; LT—lower trapezius; PM1—pectoralis major; ** significant difference within groups (*p* < 0.01); *** significant difference within groups (*p* < 0.001); # significant difference between groups (*p* < 0.05); ## significant difference between groups (*p* < 0.01).

**Figure 5:**
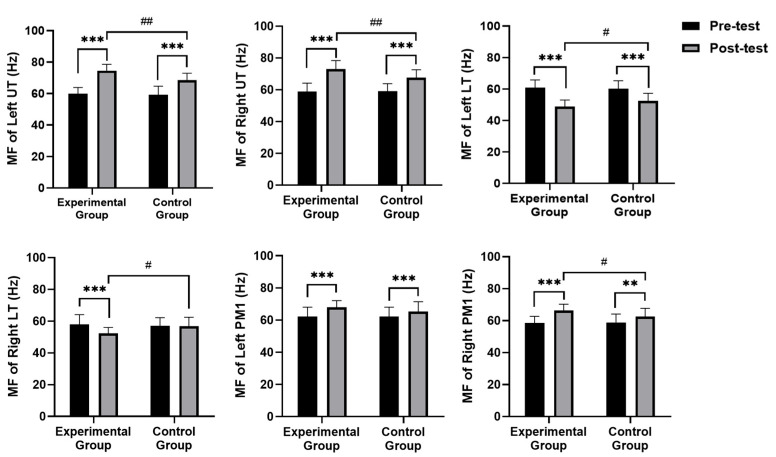
Comparison of frequency domain metrics median frequency (MF) of surface electromyography (sEMG). Note: UT—upper trapezius; LT—lower trapezius; PM1—pectoralis major; ** significant difference within groups (*p* < 0.01); *** significant difference within the group (*p* < 0.001); # significant difference between groups (*p* < 0.05); ## significant difference between groups (*p* < 0.01).

Figure 4 and Figure 5 show changes in frequency domain metrics [mean power frequency (MPF) and median frequency (MF)] of surface electromyography (sEMG) signals. While no significant group differences were observed in MPF and MF for left and right UT and LT pre-intervention (*p* > 0.05), a distinct pattern emerged post-intervention. The experimental group exhibited significantly higher MPF (left: *p* = 0.008, right: *p* = 0.032) and MF (left: *p* = 0.001, right: *p* = 0.008) in both left and right UT compared to the control group. Conversely, the experimental group displayed significantly lower MF (left: *p* = 0.037, right: *p* = 0.019) in both left and right LT, whereas no significant group differences were found in MPF. Regarding PM1, the experimental group demonstrated significantly higher MPF (left: *p* = 0.013, right: *p* = 0.005) and MF (right: *p* = 0.031) than the control group.

Within-group comparisons revealed that, compared to pre-intervention levels, both groups demonstrated significant increases in MPF and MF for both left and right UT. Both groups also experienced significant decreases in MPF (*p* = 0.000 for both sides) for both left and right LT; however, no significant changes were observed in MF. For both left and right PM1 muscles, both groups exhibited significant increases in MPF (*p* = 0.000 for both sides) and MF (left: *p* = 0.000, right: *p* = 0.002).

### 3.5. VAS and NDI Results

**Figure 6:**
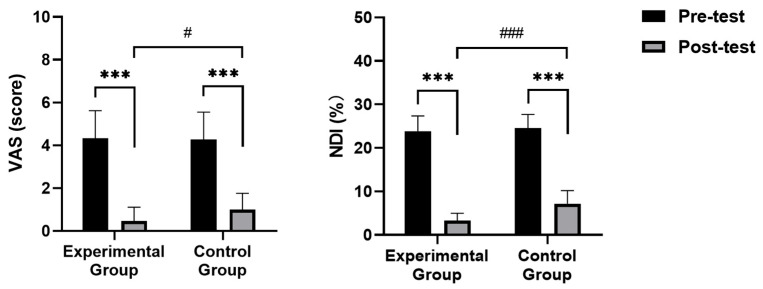
Comparison of visual analog score (VAS) and neck disability index (NDI). Note: *** significant difference within groups (*p* < 0.001); # significant difference between groups (*p* < 0.05); ### significant difference between groups (*p* < 0.001).

Figure 6 shows changes in visual analog score (VAS) and neck disability index (NDI). While no significant differences in VAS and NDI were observed between the two groups pre-intervention (*p* > 0.05), a distinct pattern emerged post-intervention. The experimental group exhibited significantly lower VAS (*p* = 0.046) and NDI (*p* = 0.000) than the control group.

Within-group comparisons revealed a significant decrease in VAS for both groups (experimental: *p* = 0.000, control: *p* < 0.05). Both groups also experienced a substantial reduction in NDI (*p* = 0.000).

## 4. Discussion

This study compared the effects of two interventions—12 weeks of Pilates training alone and Pilates training combined with fascial massage—in a UCS population based on the results of analyzing FHA, FSA, ROM, sEMG, VAS, and NDI.

This study demonstrated that both interventions significantly reduce FSA and FHA, improving posture. However, Pilates training combined with fascial massage was considerably more effective than Pilates training alone. This finding aligns with previous research. For instance, Ozturk and Unver found that a 10-week Pilates intervention decreased FSA in preschoolers, suggesting Pilates’ efficacy in correcting abnormal posture [23]. Previous studies have indicated that Pilates training can improve abnormal muscle activation, movement patterns, and postural deviations in UCS populations by enhancing core muscle activation [24]. As Jie discovered [11] through sEMG, a 12-week Pilates training can improve the fatigue resistance of the UT and PM1 muscles. Specific Pilates exercises, such as neck-pulling exercises, seated rhomboid training, single-legged pullback, deep cervical flexor stabilization, double-legged upper kick, and swimsuit, can effectively target weak and tense muscles, improving muscle balance and correcting poor posture. The myofascial chain theory emphasizes that the human body is a tensile integrity structure with bones as the scaffold, tightly connected by myofascial, ligamentous, and other tissues. Fascial massage can address muscle chain imbalances, relieve tissue pressure pain, and restore posture. Therefore, the superior efficacy of Pilates training combined with fascial massage may stem from its ability to simultaneously strengthen weak muscle groups, restore standard muscle properties, and improve muscle balance, ultimately leading to improved posture.

Our results showed that both interventions significantly enhance the ROM in cervical joints, with Pilates training and fascial massage demonstrating greater effectiveness. Vahid Mazloum et al. found that Pilates training improved pain, physical disability, and lumbar ROM in patients with chronic lower back pain [25]. Pilates training, particularly spinal flexibility exercises, can increase thoracic spine flexibility, promote spinal and pelvic alignment, and improve abnormal curvature. Additionally, fascial massage can directly target tense muscles and fascia, relieving tension, reducing muscle adhesion, and enhancing joint mobility. Combining these techniques may have synergistically improved ROM in the experimental group.

The results of sEMG demonstrated that both interventions significantly reduced fatigue in tense muscle groups (UT, PM1) while activating weak muscles (LT), with the combined intervention yielding superior results. Pilates training can improve muscle balance by enhancing the anti-fatigue ability of tense muscles and activating weak muscles. Studies like Hagberg and Kvarnström have highlighted the importance of deep neck flexor activation in reducing superficial neck muscle activity [26]. The specific Pilates exercises employed in this study targeted deep neck flexors and weak muscles, contributing to improved muscle balance. Fascial massage can increase muscle extensibility and decrease muscle stickiness, improving muscle performance. Yao Meijing demonstrated the efficacy of fascial massage in improving muscle properties in UCS populations [27]. Combining fascial massage and Pilates training may synergistically enhance muscle function and posture correction.

In addition, the combined intervention was also more effective in reducing pain levels and cervical spine dysfunction in patients with UCS compared to Pilates training alone. Previous studies have demonstrated the pain-reducing effects of Pilates, attributed to muscle stretching and improved blood flow [27,28,29]. Fascial massage can also reduce pain by improving local circulation and stimulating pain points. Studies like Lee et al. have shown the effectiveness of deep neck flexor training in reducing cervical spine dysfunction [30]. The combination of Pilates and fascial massage may have synergistically improved muscle balance, reduced pain, and enhanced joint mobility, leading to reduced cervical spine dysfunction. In summary, our results fully demonstrate the potential of combined Pilates and fascial massage as a novel therapeutic approach in clinical applications, providing new possibilities for non-pharmacological treatment for individuals with UCS.

Of course, this study also has some limitations. The sample size in this study is relatively small, and the results are limited to a specific population without age stratification. In future research, we will expand the sample size and explore the particular impact of age factors on the effect of intervention to develop more personalized treatment plans. Additionally, this study focuses on improving the physiological functions and body shapes of UCS patients but does not address the mental health dimension. Given the close connection between mental and physical health, future studies should incorporate mental health assessment indicators, such as levels of anxiety and depression, to comprehensively evaluate the health status of UCS patients.

## 5. Conclusions

This study investigated the effects of Pilates training alone and combined with fascial massage on individuals with upper cross syndrome (UCS). Both interventions significantly improved flexibility, muscle activation patterns, pain, and cervical spine dysfunction. However, Pilates combined with fascial massage demonstrated superior flexibility, muscle activation, and pain reduction outcomes. This synergistic effect is likely due to the combined benefits of muscle strengthening, increased flexibility, and improved blood circulation provided by both interventions.

## Figures and Tables

**Table 1:** Pilates exercise intervention program.

Training Phase	Exercise Program	Target Muscle	Photograph	Training Repetitions (rep/s)	Number of Training Sets (s)	Length of Rest Between Asanas (s)	Intergroup Rest Duration of Rest (s)
Warm-up exercises and fascial massage	Foam shaft and fascia gun for relaxing the fascia	Pectoralis major, pectoralis minor, upper trapezius, sternocleidomastoid, latissimus dorsi, vastus lateralis, scapularis, etc.	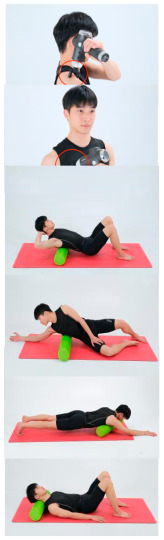	5 min			
Wake-up call (exercise)	Breathing exercises	Intercostal muscles, abdominal muscles, Pectoralis major, diaphragm	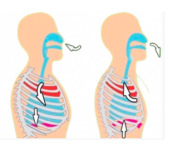	120 s			
	Shoulder flexion and extension	Pectoralis major, deltoid, latissimus dorsi, large round muscles	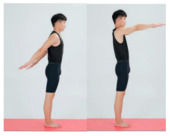	1–4 weeks :10 rep 5–8 weeks :15 rep 9–12 weeks :20 rep	1–4 weeks :1 5–8 weeks :1 9–12 weeks :1	1–4 weeks :10 5–8 weeks :10 9–12 weeks :5	1–4 weeks :20 5–8 weeks :15 9–12 weeks :10
	Squat (exercise)	Gluteus maximus, quadriceps	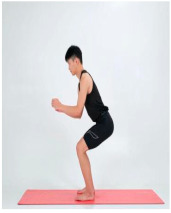	1–4 weeks :10 rep 5–8 weeks :15 rep 9–12 weeks :20 rep	1–4 weeks :1 5–8 weeks :1 9–12 weeks :1	1–4 weeks :10 5–8 weeks :10 9–12 weeks :5	1–4 weeks :20 5–8 weeks :15 9–12 weeks :10
	Standing heel lift	Gastrocnemius, Flounder	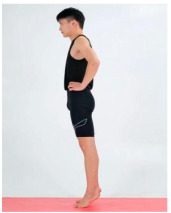	1–4 weeks :10 rep 5–8 weeks :15 rep 9–12 weeks :20 rep	1–4 weeks :1 5–8 weeks :1 9–12 weeks :1	1–4 weeks :10 5–8 weeks :10 9–12 weeks :5	1–4 weeks :20 5–8 weeks :15 9–12 weeks :10
	Pelvis clock	Rectus abdominis, internal and external abdominal obliques, transverse abdominis muscles	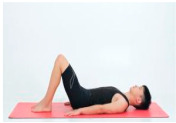	1–4 weeks :10 rep 5–8 weeks :15 rep 9–12 weeks :20 rep	1–4 weeks :1 5–8 weeks :1 9–12 weeks :1	1–4 weeks :10 5–8 weeks :10 9–12 weeks :5	1–4 weeks :20 5–8 weeks :15 9–12 weeks :10
Muscle training	Neck-pulling exercise	Deep cervical flexors (cervicalis longus, Cephalic longus, anterior rectus of the head, lateral rectus cephalicus	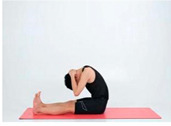	1–4 weeks :10 rep 5–8 weeks :15 rep 9–12 weeks :20 rep	1–4 weeks :1 5–8 weeks :1 9–12 weeks :1	1–4 weeks :10 5–8 weeks :10 9–12 weeks :5	1–4 weeks :20 5–8 weeks :15 9–12 weeks :10
	Seated rhomboid training	Rhomboid, middle trapezius	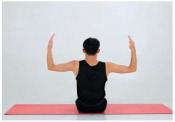	1–4 weeks :10 rep 5–8 weeks :15 rep 9–12 weeks :20 rep	1–4 weeks :1 5–8 weeks :1 9–12 weeks :1	1–4 weeks :10 5–8 weeks :10 9–12 weeks :5	1–4 weeks :20 5–8 weeks :15 9–12 weeks :10
	Single-legged pullback	Pectoralis major, abdominal muscles, hip flexor groups	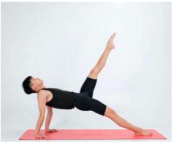	1–4 weeks :10 rep 5–8 weeks :15 rep 9–12 weeks :20 rep	1–4 weeks :1 5–8 weeks :1 9–12 weeks :1	1–4 weeks :10 5–8 weeks :10 9–12 weeks :5	1–4 weeks :20 5–8 weeks :15 9–12 weeks :10
	Deep cervical flexor stabilization	Scapularis, cephalic semispinalis	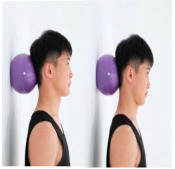	1–4 weeks :10 rep 5–8 weeks :15 rep 9–12 weeks :20 rep	1–4 weeks :1 5–8 weeks :1 9–12 weeks :1	1–4 weeks :10 5–8 weeks :10 9–12 weeks :5	1–4 weeks :20 5–8 weeks :15 9–12 weeks :10
	Double legged upper kick	Back extensor group, hamstrings	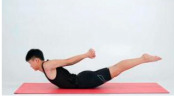	1–4 weeks :10 rep 5–8 weeks :15 rep 9–12 weeks :20 rep	1–4 weeks :1 5–8 weeks :1 9–12 weeks :1	1–4 weeks :10 5–8 weeks :10 9–12 weeks :5	1–4 weeks :20 5–8 weeks :15 9–12 weeks :10
	Freestyle stroke	Dorsal extensor group (medicine)	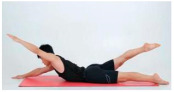	1–4 weeks :10 rep 5–8 weeks :15 rep 9–12 weeks :20 rep	1–4 weeks :1 5–8 weeks :1 9–12 weeks :1	1–4 weeks :10 5–8 weeks :10 9–12 weeks :5	1–4 weeks :20 5–8 weeks :15 9–12 weeks :10
Relaxation exercise	Baby style	Extensor digitorum longus (lower back)	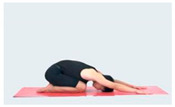	1–4 weeks :10 rep 5–8 weeks :15 rep 9–12 weeks :20 rep	1–4 weeks :1 5–8 weeks :1 9–12 weeks :1	1–4 weeks :10 5–8 weeks :10 9–12 weeks :5	1–4 weeks :20 5–8 weeks :15 9–12 weeks :10
	Inverted V-style	Pectoralis major, dorsal extensor groups, Hamstrings	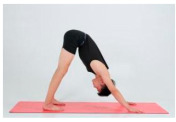	1–4 weeks :10 rep 5–8 weeks :15 rep 9–12 weeks :20 rep	1–4 weeks :1 5–8 weeks :1 9–12 weeks :1	1–4 weeks :10 5–8 weeks :10 9–12 weeks :5	1–4 weeks :20 5–8 weeks :15 9–12 weeks :10
	Spin upwards	Interosseous muscle, multifidus muscle	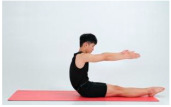	1–4 weeks :10 rep 5–8 weeks :15 rep 9–12 weeks :20 rep	1–4 weeks :1 5–8 weeks :1 9–12 weeks :1	1–4 weeks :10 5–8 weeks :10 9–12 weeks :5	1–4 weeks :20 5–8 weeks :15 9–12 weeks :10

**Table 2:** Comparison of demographic characteristics between groups.

Variable	Experimental Group		Control Group		*t*	*p*
	$\bar{X}$	SD	$\bar{X}$	SD		
Age (year)	35.87	9.09	35.40	8.24	0.147	0.884
Height (m)	1.68	0.07	1.67	0.08	0.429	0.671
Weight (kg)	68.13	7.89	65.81	7.58	0.821	0.419
BMI (kg/m^2^)	20.24	1.64	19.68	1.46	0.987	0.332
Gender (Male/Female)	6/9		8/7			

## Data Availability

Data are available upon request.
